# Predicting Perceived Stress Related to the Covid-19 Outbreak through Stable Psychological Traits and Machine Learning Models

**DOI:** 10.3390/jcm9103350

**Published:** 2020-10-19

**Authors:** Luca Flesia, Merylin Monaro, Cristina Mazza, Valentina Fietta, Elena Colicino, Barbara Segatto, Paolo Roma

**Affiliations:** 1 Associazione Novilunio Onlus, 35020 Ponte San Nicolò (PD), Italy; luca.flesia@ordinepsicologiveneto.it; 2 Department of General Psychology, University of Padua, 35131 Padua, Italy; valefietta.vf@gmail.com; 3 Department of Human Neuroscience, Sapienza University of Rome, 00185 Rome, Italy; mazzacristina87@gmail.com (C.M.); paolo.roma@uniroma1.it (P.R.); 4 Department of Environmental Medicine and Public Health, Icahn School of Medicine at Mount Sinai, New York, NY 10029, USA; colicinoelena@gmail.com; 5 Department of Political Science, Law, and International Studies, University of Padua, 35123 Padua, Italy; barbara.segatto@unipd.it

**Keywords:** COVID-19, stress, personality, public health, mental health, coping

## Abstract

The global SARS-CoV-2 outbreak and subsequent lockdown had a significant impact on people’s daily lives, with strong implications for stress levels due to the threat of contagion and restrictions to freedom. Given the link between high stress levels and adverse physical and mental consequences, the COVID-19 pandemic is certainly a global public health issue. In the present study, we assessed the effect of the pandemic on stress levels in *N* = 2053 Italian adults, and characterized more vulnerable individuals on the basis of sociodemographic features and stable psychological traits. A set of 18 psycho-social variables, generalized regressions, and predictive machine learning approaches were leveraged. We identified higher levels of perceived stress in the study sample relative to Italian normative values. Higher levels of distress were found in women, participants with lower income, and participants living with others. Higher rates of emotional stability and self-control, as well as a positive coping style and internal locus of control, emerged as protective factors. Predictive learning models identified participants with high perceived stress, with a sensitivity greater than 76%. The results suggest a characterization of people who are more vulnerable to experiencing high levels of stress during the COVID-19 pandemic. This characterization may contribute to early and targeted intervention strategies.

## 1. Introduction

SARS-Cov-2 (severe acute respiratory syndrome coronavirus 2; henceforth referred to as COVID-19) is a strain of coronavirus that can infect humans, attacking the lungs and causing symptoms ranging from those of the common cold to those of severe acute respiratory syndrome (SARS) [1]. While approximately 80% of those who are infected recover with no special treatment (i.e., they are either asymptomatic or suffer from mild pneumonia) [2], recent data have confirmed that older persons (60+ years old) [3] and persons with certain pre-existing medical conditions are more likely to develop serious respiratory distress that can lead to death (3–4% of the population) [4]. COVID-19 spreads very easily between persons and, at the time of writing, no drugs or biologics have proven effective for preventing or treating the virus [5,6].

COVID-19 was first identified in the Chinese region of Wuhan in December 2019 [6]. Between December 2019 and April 2020, the virus spread throughout the world, causing more than 5,000,000 infections and over 300,000 deaths [7]. On 11 March 2020, the World Health Organization (WHO) declared COVID-19 a pandemic [8]. To contain the number of victims and prevent the collapse of the healthcare system, most national governments imposed strict restrictions on residents’ freedom, forcing those infected with the virus to self-isolate and requiring all residents (infected or not) to stay at home and move through public areas only for reasons of absolute necessity. Among Western governments, the Italian government was the first to apply such restrictions [9].

### 1.1. Psychological Impact of the Pandemic

A few weeks after the spread of COVID-19 in China, the first scientific studies investigating the psychological impact of the outbreak highlighted a mild to severe negative psychological impact of the event within a significant proportion of the Chinese population (53.8%); specifically, this impact on mental health was reported to include symptoms of anxiety, depression, and stress [10,11]. More broadly, the literature on the impact of infectious outbreaks on mental health shows that pandemics are extremely stressful events that force people to cope with totally unexpected, ambiguous, and uncertain situations [12]. Specifically, two main aspects of pandemics have been found to affect people’s mental state. The first relates to danger (i.e., the fear of contagion), which can increase perceived threat and sometimes lead to panic, behavioral contagion, and an emotional epidemic [13,14]. The second regards the multiple and rapid changes to social, working, and familiar habits, due to self-isolation and social distancing measures [15,16,17,18,19]. The longer the duration of self-isolation, the more people experience frustration and boredom, along with concerns about infection [15].

Well-documented psychological reactions to epidemics include emotional distress, anxiety behaviors, sleeping disorders, fear, anger, depression, health concerns, a sense of powerlessness, and uncertainty [13,16,20,21,22,23,24]. Furthermore, studies examining the long-term consequences of infectious epidemics have shown that some individuals may even develop symptoms of post-traumatic stress disorder (PTSD) [12,25,26]. One review indicated that those who develop PTSD may experience the symptoms for 3 years following the end of the epidemic [15,23].

Stress is defined as an adaptive psycho-physical reaction to a physical, social or psychological stimulus, called a stressor [27]. Stress-related responses may be cognitive, emotional, behavioral, or physiological. Depending on the type, timing, and severity of exposure to a stressor, the resulting stress may become a risk factor for a number of illnesses, including those of a psychiatric or cardiovascular nature [28,29,30,31,32]. An emergency such as the COVID-19 outbreak might rightly be considered a severe stressor, as it is a new and unexpected situation with a potentially serious impact on health (experienced both personally and through loved ones) that also involves social restrictions [13]. Nevertheless, no event, in and of itself, is the precipitating cause of pathology and illness. Rather, it is the perception of stress (i.e., the degree to which one considers the event stressful) that accounts for the varying physical and mental responses to the situation [33]. In this sense, it is important to detect vulnerable persons early, and to promote effective preventive programs in order to treat such persons rapidly and limit negative psychological outcomes. The identification of psycho-social risk and resilience factors for psychological distress during the COVID-19 emergency comprises a significant step in this direction [34].

### 1.2. Risk and Protective Factors for Psychological Outcomes During the Pandemic

To date, studies on the psychological impact of COVID-19 have mainly focused on the role of sociodemographic variables (e.g., gender, age, education level, and social connections) in moderating reactions to the outbreak [10,11,24,35,36]. The identified sociodemographic risk factors for psychological distress include gender (female), age (18–30 or 60+ years old), student status, education level, perception of the public health system, specific physical symptoms (e.g., coryza, cough, sore throat, headache), and a low reported level of health [10,24,35]. For Chinese students, living in an urban area, having a stable family income, living with parents, and having good social support were also found to protect against anxiety [11].

In addition to investigating sociodemographic factors, many studies have also outlined the role of certain dispositional traits in modulating responses to stressful events. However, these constructs have been poorly investigated in relation to the psychological impact of epidemics and, specifically, COVID-19. More generally, research has shown how individual differences, including dispositional traits, can explain life outcomes [37]. According to the theory of traits (or dispositional theory), individual differences may be explained by certain predispositions (traits), which are expressed in a relatively stable way across situations and time. Traits comprise a person’s manner of thinking, feeling, perceiving, and relating to others [37]. Based on these considerations, dispositional traits might play a relevant role in predicting perceived stress in relation to COVID-19.

Coping is one of the most widely studied dispositional traits, and it has been found to be significant in modulating responses to stressful events. Coping is defined as the effort to solve personal and interpersonal problems in an attempt to master, minimize, or tolerate stress and conflict [38]. Distinct coping strategies have been found to be differentially associated with specific emotional responses [39], physiological stress responses [40], and self-efficacy [41,42]. One investigation into the role of coping strategies during a virus outbreak (2009 H1N1 flu pandemic) found coping style to influence the perceived risk of contagion and vaccination intentions among Canadian adults [43]. Another study, based in Singapore, found coping strategies to be associated with post-traumatic outcomes within visitors to community health care services during the national outbreak of SARS [23].

As regards emotional self-regulation and adaptation to the world, self-control may represent a significant protective factor. There is empirical evidence that people with high dispositional self-control have better psychological adjustment and impulse control [44]; this suggests that good self-control may mitigate the influence of a negative environment. Similarly, perceived control over life outcomes has been shown to be positively associated with well-being and health-related quality of life, and negatively associated with emotional distress, in the context of stressful events [45,46]. In this regard, locus of control [47] is a relevant concept, describing the degree to which an individual believes that they have control over the outcome of life events, as opposed to feeling that their life is subject to external forces beyond their control. Finally, among the many individual difference variables that might influence reactions to COVID-19, personality traits merit significant attention. Several studies have highlighted an association between the Big Five personality traits [48] and various health behaviors, such as sedentary behavior [49], sexual health behavior [50], physical activity [51], and alcohol consumption [52].

The first aim of the present study was to investigate the impact of the COVID-19 pandemic and the related government-imposed restrictions on perceived stress in a Western country (i.e., Italy). As reported above, most studies on the psychological impact of COVID-19 have related to the Chinese population. However, countries differ from one another in many important aspects (i.e., social, cultural, political, and economic aspects, to name only a few); consequently, psychological responses may also vary between contexts and communities, revealing unique qualitative and quantitative psychological reactions and psychological needs.

**Hypothesis** **1** **(H1).** *Our sample of Italian adults, collected during the COVID-19 outbreak, would show higher levels of perceived stress compared to Italian normative values.*

The second aim of the study was to confirm the role of certain sociodemographic factors in modulating stress responses to the COVID-19 pandemic, as reported in the recent literature.

**Hypothesis** **2** **(H2).** *(a) Participants who were female, younger, and students, with a lower level of education and lower income, would report higher levels of stress, and (b) participants who were living with others would report lower levels of psychological distress.*

The third aim of the work was to investigate the association between certain stable psychological traits and psychological distress relating to the current situation. To this end, participants were tested for coping strategies, self-control, locus of control, and select personality traits.

**Hypothesis** **3** **(H3).** *(a) Participants with positive coping strategies, higher levels of self-control, an internal locus of control, and higher levels of emotional stability would report lower levels of stress, and (b) participants with negative coping strategies, lower levels of self-control, an external locus of control, and lower levels of emotional stability would report higher levels of stress.*

Finally, with the goal of anticipating persons in need of treatment and improving the targeting and overall effectiveness of preventive programs, we aimed at developing machine learning models to predict individual psychological responses to the COVID-19 pandemic, based on sociodemographic and psychological variables with maximal sensitivity in classifying subjects with high versus low levels of perceived stress.

To summarize, the study was novel in the following two respects: first, it considered the role of not only sociodemographic variables, but also stable psychological traits, as predictors of a stressful reaction to COVID-19; and second, it leveraged machine learning techniques to identify people at the greatest risk of developing severe and negative psychological outcomes due to the pandemic.

## 2. Materials and Methods

### 2.1. Measures

To test the abovementioned hypotheses, we implemented a cross-sectional study. Using Google Forms, we designed an ad hoc online questionnaire to collect data on participants’ stress reactions to COVID-19, demographical variables, and psychological traits. The questionnaire also assessed the following sociodemographic factors: gender, age, education, number of family members and/or others living in the household, monthly household income, and student status. Subsequently, we administered five standardized questionnaires, as follows:

- The Italian Version of the 10-item Perceived Stress Scale (PSS-10; Cronbach’s alpha = 0.74) [53]. The PSS-10 is a frequently used psychological instrument to measure perceived stress [54]. Respondents are asked to answer 10 questions pertaining to the frequency of experiences of stressful situations during the last month on a five-point scale ranging from 0 (never) to 4 (very often) [55,56]. Example items include “In the last month, how often have you been upset because of something that happened unexpectedly?” Higher scores indicate higher levels of perceived stress. Moreover, in the present study, the score corresponding to 1.5 SD above the Italian normative score [53] was used as a cut-off to divide participants into two classes: low perceived stress (males: PSS-10 score <24.35; females: PSS-10 score <24.55) and high perceived stress (males: PSS-10 score ≥24.35; females: PSS score-10 ≥24.55);
- The Italian Shortened Version of the Coping Orientations to the Problems Experienced (COPE-NVI-25; Cronbach’s alpha of factors range 0.68–0.95) [57]. The COPE-NVI-25 is a multi-dimensional inventory that assesses individual differences in coping styles. It is comprised of 25 items, which are rated on a 4-point scale ranging from 1 (I usually don’t do this at all) to 4 (I usually do this a lot) [58]. The instrument includes five subscales corresponding to five different coping styles: social support, avoidance strategies, positive attitude, problem solving, and turning to religion [59]. An example item is “I admit to myself that I can’t deal with it, and quit trying” (avoidance strategies). A higher score on a particular subscale indicates a greater use of that specific coping strategy.
- The Italian translation of the Brief Self-Control Scale (BSCS; Cronbach’s alpha = 0.85) [44]. The BSCS measures individual differences in dispositional capacity for self-control. The scale is comprised of 13 items that are rated on a five-point scale ranging from 1 (not at all) to 5 (very much). An example item is “I do certain things that are bad for me, if they are fun.” Higher scores on the BSCS indicate a greater capacity for self-control, and they are also correlated with better psychological adjustment, interpersonal skills, and emotional responses [44];
- The Italian Short Version of the Locus of Control (LOC) Scale [60]. This 20-item questionnaire is used to measure generalized expectancies relating to an internal versus external locus of control, rated via dichotomous options (“Yes” vs. “No”), similar to Rotter’s original Internal–External Locus of Control Scale [47]. An example item is “To do well in life, luck is more important than commitment.” Respondents with an internal locus of control (i.e., a high score on the internal LOC scale) tend to attribute life outcomes—and general life events—to their own behavior, whereas those with a prevalent external locus of control (i.e., a high score on the external LOC scale) tend to attribute life events to fate, others, or external causes beyond their control [47,61];
- The Italian Version of the 10-item Big Five Inventory (BFI-10; Spearman–Brown coefficients ≥0.50) [62]. The BFI-10 assesses personality traits according to the five-factor approach [63]. It is comprised of 10 items rated on a five-point scale ranging from 1 (strongly disagree) to 5 (strongly agree), measuring five dimensions of personality, which are extraversion, agreeableness, conscientiousness, neuroticism (or, if reversed, emotional stability), and openness [64]. An example item is “I see myself as someone who is outgoing, sociable” (extraversion). The higher the score on a particular subscale, the more that specific dimension represents a characteristic trait of the respondent’s personality.

The complete list of 18 variables that were extracted from the responses to the questionnaire is provided in the Supplementary Materials. The present research was designed in accordance with the Declaration of Helsinki and approved by the ethics committee for psychological research at the University of Padova (protocol number 3576, unique code 189B46FE116994F1A8D1077B835D83BB).

### 2.2. Participants and Procedure

Data were collected during the period of 20–31 March, 2020. Participants were recruited online through an invitation posted on social media (Facebook and WhatsApp). This approach of online recruitment was selected primarily due to the lockdown situation, which prevented us from collecting data in the laboratory. According to the aim of the study, it was necessary for us to capture the psychological state of participants at the time of the pandemic; thus an a posteriori study would not have provided useful and reliable information.

Participants were invited to complete an anonymous online questionnaire to report their personal experiences with the COVID-19 emergency and their mental state. The inclusion criteria were the following: (a) living in Italy at the time of data collection and (b) being aged 18+ years (18 years is the legal age in Italy, defined by the capacity to act and be emancipated). Participation was voluntary. All participants were required to read and provide informed consent before beginning the online questionnaire. They received no compensation for their participation. In total, 2072 volunteers took part in the study. Of these, 19 were excluded on the basis that they responded to the questionnaire twice (we kept only their first response). Thus, the final sample was comprised of 2053 participants, of whom 1555 were female, 480 were male, and 18 were reported as “other.” The participants’ average age was 35.81 (*SD* = 13.19; range: 18–83) and their average education level was 15.35 years (*SD* = 3.43; range: 5–21). A more detailed description of the sample’s demographic characteristics is provided in the Supplementary Materials. It has been calculated that a sample size of 2053 is sufficiently large to achieve at least a statistical power (1-β) = 0.90 in a linear multivariable regression analysis involving 18 predictors, given a significance level α = 0.05 and an effect size of 0.356 [65]. Data are provided in the Supplementary Materials.

### 2.3. Data Analysis Methodology

#### 2.3.1. Statistics

Data analysis was conducted using the JASP software [66]. A single sample *t*-test (*t*, two-sided) was performed in relation to the PSS-10 score, in order to determine whether the sample’s true mean (*μ*) was statistically different from that of the known population (*m*_0_). A multivariable regression analysis was run to investigate the relationship between the PSS-10 score and the independent variables that were hypothesized to impact the level of perceived stress. The collinearity assumption was checked prior to running the model, using the tolerance and variance inflation factor (VIF). As a rule of thumb, if VIF >10 and tolerance <0.1, the assumption is greatly violated, whereas if VIF >1 and tolerance <0.2, the model may be biased [67]. The results indicated that the collinearity assumption was not violated by any of the independent variables entered in the regression model. The analysis was performed using the stepwise variable selection method, which identified predictors with a significant (*p* < 0.05) individual association with the outcome (PSS-10 score). The results were reported using unstandardized coefficients, as recommended by Friedrich [68].

#### 2.3.2. Classification Models

Recently, researchers in different scientific fields, including the clinical and social sciences, have emphasized the utility of focusing on prediction, rather than explanation, during data analysis [69,70,71,72]. This increased attention to predictive models may be largely attributed to the significant spread of machine learning (ML)—a branch of artificial intelligence that trains algorithms on data samples (i.e., training sets) in order to make predictions on completely new data (i.e., test sets) without being explicitly programmed to do so [73]. As regards psychology, ML techniques have been shown to be particularly useful for predicting human behavior, including high-risk behavior; thus, they may be applied to improve the effectiveness and targeting of preventive programs and interventions [74]. In brief, ML models are capable of predicting the behavior of individual subjects, allowing greater attention to be paid to those considered most critical [69].

In the present study, ML algorithms were trained on psycho-social data to identify subjects who were more likely to present high levels of perceived stress during the COVID-19 emergency, and who were consequently at the greatest risk of developing psychological symptoms, including those of PTSD. For this purpose, participants were split into two classes: high perceived stress and low perceived stress. The high perceived stress class included participants with a PSS-10 score of more than 1.5 *SD* above the Italian population mean (*n* = 393) for men and women, respectively. Conversely, the low perceived stress class included participants whose PSS-10 did not exceed 1.5 *SD* above the Italian normative value (*n* = 1642). It should be noted that participants who reported their gender as “other” (*n* = 18) were excluded from this analysis, as the Italian normative values were available for males and females only [53].

As ML models are built to fit particular data, it is important to test how each model fits new (i.e., unseen) data. For this reason, part of the data (the training set) is generally used to train and validate the model, while another part (the test set) is used to test the model’s accuracy on new examples [73,75]. This procedure guarantees the model generalization and increases the replicability of the results [76,77]. In the present study, 20% [73,75] of the participants were randomly chosen and retained as the test set. Accordingly, the training set consisted of 1628 participants (314 with high perceived stress and 1314 with low perceived stress), and the test set consisted of 407 participants (79 with high perceived stress and 328 with low perceived stress).

In the first step, feature selection was performed to remove redundant and irrelevant features and to increase model generalization by reducing overfitting and noise in the data [78]. A good strategy for feature selection is to identify the subset of features that are highly correlated with the class to predict, but not correlated with each other [78]. This procedure was performed in the present study using the correlation-based feature selector (CFS) in the WEKA 3.9 software [79].

The problem of class imbalance was addressed while running the classification algorithms. The ratio between participants with high perceived stress and those with low perceived stress was approximately 1:5. As ML methods work best with balanced datasets, it is necessary to account for any class imbalance, especially when training examples are limited—a condition that is frequently met by datasets in health and clinical psychology [80]. At the same time, it is equally important for ML models to be built on samples that are representative of the population, reflecting real distribution [80].

One strategy to overcome these two limitations consists of altering the relative costs associated with misclassifying the minority and majority classes, in order to compensate for the class imbalance [81]. In the present study, ML algorithms were set in such a way that any algorithmic error made in classifying the minority class (high perceived stress) was weighted four times more than any error in classifying the majority class (low perceived stress). This cost-modifying strategy has been shown to provide better results than other methods in addressing the class imbalance problem [81]. Moreover, it should be noted that, for the goal of the present task, it was more beneficial to minimize false negatives than to minimize false positives (i.e., to have a model with high sensitivity rather than high specificity). In other words, it was more important to identify people who were truly at risk than to avoid misclassifying people who were not truly at risk.

ML models were trained and validated on the training sample (*n*= 1628) through a 10-fold cross-validation procedure using the WEKA 3.9 software [79]. The different algorithms (i.e., logistic regression [82], support vector machine (SVM) [83], Naïve Bayes [84], random forest [85]) were chosen as representatives of different classification strategies, to ensure that the results would be stable across classifiers and not dependent on specific model assumptions (details on the parameters of the ML classifiers are reported in the Supplementary Materials). K-fold cross-validation is a resampling procedure that seeks to reduce the variance in model performance relative to the performance that may be obtained from a single training set and a single test set. The procedure consists of portioning the sample into k subsets (i.e., folds; in the present study, k = 10), and using k-1 (i.e., 9) subsets to train the model and the remaining subset to validate the model’s accuracy. This is repeated k (i.e., 10) times [86]. The final model metrics are obtained by averaging the metrics obtained in all validation subsets. In the present study, the models developed from the 10-fold cross-validation procedure were tested on the test sample (*n* = 407).

## 3. Results

The main results of the data analysis are reported in this section. A more complete descriptive analysis of each variable, including the composition of high perceived stress versus low perceived stress samples, is reported in the Supplementary Materials.

### 3.1. Perceived Stress in Response to the COVID-19 Emergency

The average PSS-10 score of the entire sample was 18.81 (*SD* = 6.25). Analyzing the responses of males and females separately (note that participants who reported a gender of “other” were excluded from this analysis due to a lack of normative data), males obtained an average score of 16.71 (*SD =* 6.91) and females obtained an average score of 19.44 (*SD* = 6.79). To determine whether the sample mean statistically differed from that of the Italian normative population (males: average = 15.2, *SD* = 6.1; females: average = 16.3, *SD* = 5.5) [53], a one-sample *t*-test was run separately for each gender. Statistically significant results emerged for both males (*t*_(479)_ = 4.79, *p* < 0.001, *d* = 0.22, 95% CI for Cohen’s *d* (0.13, 0.31)) and females (*t*_(1554)_ = 18.21, *p* < 0.001, *d* = 0.46, 95% CI for Cohen’s *d* (0.41, 0.510)). The PSS-10 items showing the greatest increase in participants with high perceived stress (1.5 *SD* above the population mean) were those related to loss of control over one’s life (*M* = 3.09, *SD* = 0.80) and frequently feeling nervous or stressed (*M* = 3.51, *SD* = 0.60) (see Supplementary Materials for an item-by-item analysis).

### 3.2. The Role of Sociodemographic Variables in Predicting Perceived Stress

A first multiple regression analysis was run, including sociodemographic variables that have been shown to potentially impact the level of perceived stress during a pandemic [10,11,24,35,36]. The PSS-10 score was set as the dependent variable, while gender (male), age, education, monthly income, number of family members, and student status (student) were entered as covariates. The final model accounted for a significant proportion of the variance in the level of perceived stress (*R*^2^ = 0.103, adjusted *R*^2^ = 0.101, *F*-change_(1,2029)_ = 12.009, *p* < 0.001). All of the aforementioned variables, with the exception of student status, were found to contribute to the level of perceived stress. Results are reported in Table 1.

### 3.3. The Role of Stable Psychological Traits in Predicting Perceived Stress

To better understand the role of stable psychological traits in predicting the level of perceived stress (PSS-10 score), a second multiple linear regression was run, adding to the previous model the scores of the five coping styles measured by the COPE-NVI-25 (COPE positive, COPE problem, COPE avoidance, COPE religion and COPE support), the BSCS total score, the internal LOC score, and the scores for the five personality traits measured by the BFI-10 (BFI-10 agreeableness, BFI-10 conscientiousness, BFI-10 emotional stability, BFI-10 extraversion and BFI-10 openness). This second model accounted for a larger proportion of the variance in the level of perceived stress (*R*^2^ = 0.356, adjusted *R*^2^ = 0.352, *F*-change_(1,2022)_ = 5.908, *p* < 0.05) compared to the previous model. BFI-10 emotional stability, COPE positive, age, BCSC total score, gender (male), COPE avoidance, internal LOC, number of family members, COPE support, monthly income, and BFI-10 conscientiousness were identified as significant predictors of the level of perceived stress during the COVID-19 epidemic (see Table 2). Education, COPE religion, COPE problem solving, BFI-10 agreeableness, BFI-10 extraversion and BFI-10 openness were excluded.

### 3.4. Machine Learning Classification Models

Ultimately, the 18 questionnaire variables were considered predictors of perceived stress. The entire list of predictors, along with their descriptions, is provided in the Supplementary Materials. Of these 18 variables, the following 7 were identified as the best set of predictors using correlation-based feature selection: age, monthly income, COPE avoidance, COPE positive, BSCS total score, BFI-10 emotional stability, and BFI-10 agreeableness. Using these predictors, ML algorithms were trained and tested according to the procedure described in the “Data Analysis” section. Classification results for the test set are reported in Table 3, which quantifies predictive performance according to the following metrics: receiver operating characteristic curve (ROC) area, precision, recall and F-measure (F1 score). It is worth noting that the classifiers showed an ROC area ranging from 0.70 to 0.78 in the test set. However, the random forest algorithm highlighted the lower sensitivity (recall) of the high perceived stress class compared to the other classifiers, making it a weaker model for the purposes of prediction.

## 4. Discussion and Conclusions

The present study measured the impact of the COVID-19 emergency on perceived levels of stress, taking into account sociodemographic variables and stable psychological traits. The results confirmed that participants perceived the COVID-19 crisis as a stressful experience; in the present sample, the level of perceived stress was higher than that of the general population in a non-emergency condition. Indeed, almost 20% of the sample scored above the results from the normative data on measures of perceived stress. These results are in line with the findings of recent studies on the psychological impact of COVID-19 [10,11,87] and the international literature on epidemic outbreaks [13]. The mean values of the single items of the PSS-10 suggest that, in addition to nervousness and stress, feelings of being unable to control one’s personal life accounted for the majority of participants’ perceived stress. This suggests that the unpredictability and uncontrollability of the pandemic may play a significant role in determining levels of perceived stress during the crisis. Moreover, it may reflect participants’ attitudes toward the significant lifestyle changes demanded of them due to the lockdown and other restrictive measures.

As regards sociodemographic variables, the results suggest that the female gender is associated with higher levels of stress. This is consistent with the literature indicating gender differences in the psychological response to COVID-19 [24,88] and other epidemics [89]; it is also in line with the normative data for the general population. Consistent with other studies (20,30), the present study found an association between higher incomes and lower levels of perceived stress. One explanation for this is that higher incomes might be related to less concern about the economic effects of self-isolation and/or with more comfortable housing solutions (e.g., larger living spaces, access to outdoor spaces (such as gardens), and access to leisure activities). Moreover, people with higher incomes may be more likely to perform work that can easily and fully transition to the online environment, thereby reducing some sources of stress.

In the present sample, older age was found to be associated with lower levels of stress. This finding might appear surprising, since it contradicts both the results of studies on the Chinese population [24,35] and the association between older age and higher COVID-19 mortality. However, the result is in line with recent Italian data [87]. Several studies have indicated age-related differences in coping and locus of control, with older adults presenting greater self-control and emotional self-regulation relative to younger adults [90,91,92]. Considering the current pandemic, older people may be more used to staying at home, so their daily routines might be less impacted by mandatory self-isolation measures. Data from previous investigations on age differences in stress responses to the SARS epidemic reflect inconsistencies [93,94], but sociopolitical and cultural aspects, such as differences in elder care services and policies, might account for these discrepancies.

The present study did not find education to be a significant predictor of the level of perceived stress. The large percentage of highly educated participants in the sample might partially explain this finding. However, prior research on this subject has generated mixed results—recent studies on the Chinese population [24,35] have found that education does not seem to affect mental health, while data from a Spanish sample [88] and from previous studies on psychological adjustment to SARS [95] have confirmed an association between a higher level of education and better mental health. The present study also found that living alone or with few family members was a protective factor against perceived stress. We might argue that this condition both conveys a sense of protection from contagion and offers continuity with pre-epidemic economic and social conditions. Moreover, when cohabiting with family members, concern for loved ones might contribute to increasing perceived stress.

As regards psychological variables, emotional stability was found to be an important protective factor. According to the five-factors model [63], people with high emotional stability remain calm in response to stressful situations, and view problems in proportion to their importance. As a result, they tend to worry less about problems than do people with low emotional stability [96]. Many studies have found that emotional stability is able to buffer stress responses to adverse events [97,98]. In the present study, conscientiousness and agreeableness were found to predict psychological distress. Generally speaking, individuals who score high on agreeableness tend to dislike conflict and be less suspicious of others; generally, they seek to pacify and mediate. In this sense, agreeable people might be more flexible and accepting when faced with unexpected and undesired situations, such as restrictions and changes to daily routines. Conscientious people are more likely to perceive lower levels of stress (see correlation analysis). According to the literature, they are more aware of their actions and tend to exhibit more goal-oriented behavior. In this sense, in times of self-isolation, conscientious people might have a greater tolerance for frustration and imposition relative to less conscientious people, who might engage in more impulsive behavior [96]. Moreover, previous research has indicated that conscientiousness may influence adaptive behavior, especially in health-related programs [99,100].

The present study found higher levels of dispositional self-control to predict lower levels of psychological distress in response to the COVID-19 emergency. Personal self-control skills may play a role in determining tolerance to restrictions to personal freedom during self-isolation. This result further suggests that self-regulatory processes may have a strong influence on responses to the outbreak. The results regarding dispositional self-control are consistent with those relating to the emotional stability trait of the Big Five model. In fact, these dimensions are often correlated [101], and this specific pattern may indicate the importance of personal skills, such as the ability to remain calm, and maintain emotional balance and a sense of acceptance. In this sense, practices that enhance emotional stability and acceptance, such as mindfulness, could be useful in reducing the stressful impact of the emergency [102,103]. The results concerning coping styles are also in line with this. Besides confirming the protective effect of functional coping styles and the adverse impact of dysfunctional coping styles, the results of the present study suggest that people who use a positive attitude as a coping strategy may be much less likely to experience psychological distress during the present emergency. Such persons may appraise the emergency as a unique opportunity, and feel less need for psychological support. In contrast, the present findings suggest that people who use avoidance strategies may be more likely to experience higher levels of stress during the emergency. These results are consistent with the findings of previous investigations into the relation between coping style and response to an epidemic [23,43,104,105].

The results relating to dispositional locus of control indicate that people with an internal locus of control may be less likely to feel stressed. Again, these results suggest that the more people are inclined to confidently rely on themselves, the better they will cope with uncertainty and change. Several studies have indicated an association between an internal locus of control and self-efficacy and emotional stability [106,107]. Furthermore, previous studies have found a relation between an internal locus of control and the positive appraisal of an emerging infectious disease outbreak [93]. It could be hypothesized that people with an internal locus of control interpret self-isolating as something that they determine and enact for themselves as a protective behavior, rather than something that is imposed on them; this might account for their lower levels of perceived stress.

Overall, the results of the present study identify some population subgroups that may be more vulnerable to experiencing stress during the COVID-19 emergency. Specifically, a set of seven psycho-social variables may identify a high percentage of people experiencing high stress during the COVID-19 pandemic, with sensitivity approaching 0.759 (ROC area of predictive models ranging between 0.70 and 0.78). According to this model, we may develop targeted preventive interventions. Furthermore, self-regulatory skills (including emotional stability, an internal locus of control, and self-control) were shown to be a protective factor, indicating the importance of raising awareness of these skills during the emergency and offering training and education to increase personal abilities in these areas (e.g., mindfulness programs).

The present research aimed at improving our understanding of the possible risk and protective factors for high perceived stress during the COVID-19 outbreak. It is worth noting that all data were collected from an Italian population. Therefore, the findings were inevitably influenced by specific contextual and socio-cultural aspects. Further investigations involving people of different ethnicities and residents of other countries would deepen our understanding of the generalizability of these results, and the effective influence of psychological traits. In this regard, the open access data reported in the Supplementary Materials may contribute to facilitating comparisons between ethnicities, countries, and specific traumatic events.

Some further limitations should be considered when interpreting the findings of this research. First, participants were recruited via an online link posted on social networks. While online recruitment guarantees large samples, it does not guarantee sample representativeness. For this reason, very vulnerable groups, such as homeless or low-income persons, may not be well represented in this study. Similarly, the average age of the sample was young, predominantly female, and largely well educated, as indicative of a sample that is more likely to participate in an online survey. Second, the use of self-report measures did not enable us to verify the reliability of the responses, or to ensure that participants correctly understood the questions. Future research should aim at overcoming these shortcomings.

Finally, future research should also investigate the interplay and mutual interrelationship between protective and risk factors, to improve the targeting and overall effectiveness of preventive programs and interventions. Indeed, the literature suggests that, during a pandemic, it is extremely important for people to sustain their use of psychological services, either online or in the context of social distancing [108,109]. This is particularly essential for those who are more vulnerable to experiencing high levels of stress, and it is important that we ensure that such persons can access timely and high-quality psychological services in order to prevent the development of chronic outcomes, including PTSD.

## Figures and Tables

**Table 1:** Multiple Linear Regression Model Predicting the Level of Perceived Stress Based on Sociodemographic Variables.

						95% CI	
	ΔR^2^	Unstandardized Coefficients (B)	S.E.	*t*	*p*	Lower Bound	Upper Bound
(Intercept)		25.342	0.987	25.665	4.845e^−126^	23.405	27.278
Age	0.067	−0.121	0.012	−10.506	3.540e^−25^	−0.143	−0.098
Gender (male)	0.024	−2.406	0.345	−6.968	4.321e^−12^	−3.084	−1.729
Education	0.004	−0.081	0.044	−1.842	0.066	−0.168	0.005
Household members	0.003	0.453	0.129	3.500	4.748e^−4^	0.199	0.706
Monthly income	0.005	−0.463	0.134	−3.465	5.403e^−4^	−0.725	−0.201

Note: Root Mean Square Error (RMSE) = 6.56. Analysis of Variance (ANOVA) F_(5,2029)_ = 46.65, *p* < 0.001.

**Table 2:** Multiple Linear Regression Model Predicting the Level of Perceived Stress Based on Sociodemographic Variables and Stable Psychological Traits.

						95% CI	
	ΔR^2^	Unstandardized Coefficients (B)	S.E.	*t*	*p*	Lower Bound	Upper Bound
(Intercept)	0.000	36.238	1.540	23.531	<0.001	33.218	39.258
BFI-10 emotional stability	0.221	−0.959	0.067	−14.346	<0.001	−1.091	−0.828
COPE positive	0.047	−2.357	0.211	−11.195	<0.001	−2.770	−1.944
Age	0.033	−0.070	0.010	−6.814	<0.001	−0.090	−0.050
BSCS total score	0.018	−0.127	0.019	−6.646	<0.001	−0.165	−0.090
Gender (male)	0.016	−1.883	0.302	−6.228	<0.001	−2.476	−1.290
COPE avoidance	0.007	1.411	0.328	4.307	<0.001	0.768	2.053
Household members	0.003	0.406	0.108	3.751	<0.001	0.194	0.619
BFI-10 conscientiousness	0.003	0.276	0.098	2.809	0.005	0.083	0.469
COPE support	0.002	0.519	0.168	3.087	0.002	0.189	0.849
Monthly income	0.002	−0.283	0.111	−2.543	0.011	−0.502	−0.065
Internal LOC	0.002	−0.145	0.060	−2.431	0.015	−0.262	−0.028

Note: RMSE = 5.57. ANOVA F_(12,2022)_ = 101.49, *p* < 0.001. BFI-10 = 10-item Big Five Inventory; COPE = Coping Orientations to the Problems Experienced; BSCS = Brief Self-Control Scale; LOC = Locus of Control.

**Table 3:** Metrics of the ML Models Tested on the 407 New Participants (Test Set).

Algorithm	ROC Area	Class	Precision	Recall	F-Measure
Logistic	0.782	High perceived stress	0.349	0.759	0.478
		Low perceived stress	0.919	0.659	0.767
SVM	0.697	High perceived stress	0.337	0.747	0.465
		Low perceived stress	0.914	0.646	0.757
Naïve Bayes	0.776	High perceived stress	0.361	0.709	0.479
		Low perceived stress	0.909	0.698	0.790
Random forest	0.776	High perceived stress	0.438	0.532	0.480
		Low perceived stress	0.881	0.835	0.858

Note: ROC = receiver operating characteristic curve; SVM = support vector machine.

## Supplementary Materials

The following are available online at https://www.mdpi.com/2077-0383/9/10/3350/s1, Table S1: Descriptive statistics, Table S2: Item by item analysis of the PSS-10, Table S3: List of predictors, Table S4: Details on ML classifiers parameters.
