# Supplementary material for: Predicting Perceived Stress Related to the Covid-19 Outbreak through Stable Psychological Traits and Machine Learning Models
Source: J Clin Med. 2020 Oct 19;9(10):3350. doi: 10.3390/jcm9103350 (PMC7603217; doi:10.3390/jcm9103350)
Supplement: Supplementary file 1 [file jcm-09-03350-s001.pdf]

Predicting Perceived Stress Related to the Covid-19 Outbreak through Stable Psychological Traits and Machine Learning Models

SUPPLEMENTARY MATERIALS

Table S1: Descriptive statistics. The table reports the descriptive statistics of the sample for the 18 analyzed variables. The first column indicates the analyzed variable. The second column reports the composition of the entire sample for the considered variable. The third and the fourth columns contain, for each considered variable, the composition of the sample of the participants with low perceived stress and with high perceived stress. Finally, the last four columns report the results of the correlation between the considered variable and the 10-item Perceived Stress Scale (PSS-10) score, including *p*-value and 95% CI values.

	**All participants**	**Low perceived stress †**	**High perceived stress (>1.5 DS) †**	**Correlation with PSS-10**		**95% CI**	
				***r*/*r*$_{pb}$**	***p***	***Lower bound***	***Upper bound***
**N**	2053	1642 (79.98%)	393 (19.14%)				
**Age**	Avg. age = 35.81 (SD = 13.19)<br>Risk age (≥ 60 years old) = 6.43%	Avg. age = 37.07 (SD = 13.42)<br>Risk age (≥ 60 years) old = 7.55%	Avg. age = 30.88 (SD = 10.97)<br>Risk age (≥ 60 years old) = 2.04%	r= –0.26***<br>r$_{pb}$= –0.15***	$1.458e^{-33}$<br>$3.462e^{-12}$	–0.30<br>–0.20	–0.22<br>–0.11
**Gender**	Male= 23.38%<br>Female= 75.74%<br>Other= 0.88%	Male= 25.34%<br>Female= 74.67%	Male= 16.29%<br>Female= 83.72%	r$_{pb}$= –0.17*** †	$3.123e^{-14}$	–0.21	–0.13
**Education level**	Avg. = 15.36 (SD = 3.43)<br>5 years = 0.05%<br>8 years = 7.16%<br>13 years = 38.19%<br>16 years = 19.83%<br>18 years = 21.97%<br>More than 18 years = 12.81%	Avg. = 15.47 (SD = 3.52)<br>5 years = 0.06%<br>8 years = 7.67%<br>13 years = 35.99%<br>16 years = 19.06%<br>18 years = 23.08%<br>More than 18 years = 14.13%	Avg. = 14.94 (SD = 3.00)<br>5 years = 0.00%<br>8 years = 5.09%<br>13 years = 46.82%<br>16 years = 22.14%<br>18 years = 18.32%<br>More than 18 years = 7.63%	r= –0.05*	0.036	–0.09	–0.003
**Number household members**	Avg. = 2.91 (SD = 1.23)	Avg. = 2.87 (SD = 1.22)	Avg. = 3.05 (SD = 1.25)	r= 0.13***	$3.502e^{-9}$	0.09	0.17

**Student status**	Student = 24.50%	Student = 20.71%	Student = 39.19%	r_{pb}= 0.19***	$4.488e^{-18}$	0.15	0.23
**Monthly household income**	(1) <500 = 3.17%<br>(2) 500-1000 = 8.87%<br>(3) 1000-2000 = 34.88%<br>(4) 2000-3000 = 30.20%<br>(5) 3000-4000 = 14.81%<br>(6) >4000 = 8.09%	<500 = 2.62 %<br>500-1000 = 8.04 %<br>1000-2000 = 34.96%<br>2000-3000 = 30.69 %<br>3000-4000 = 15.29 %<br>>4000 = 8.40 %	<500 = 5.34 %<br>500-1000 = 12.72 %<br>1000-2000 = 33.59 %<br>2000-3000 = 28.75 %<br>3000-4000 = 12.98 %<br>>4000 = 6.62 %	r= –0.08***	$3.062e^{-4}$	–0.12	–0.04
**COPE-NVI-25**	Functional coping<br>Avg. = 5.65 (SD = 1.04)	Functional coping<br>Avg. = 5.76 (SD = 0.99)	Functional coping<br>Avg. = 5.19 (SD = 1.11)	r= –0.32***	$5.326e^{-49}$	–0.36	–0.28
	Positive attitude<br>Avg. = 2.99 (SD = 0.64)	Positive attitude<br>Avg. = 3.07 (SD = 0.61)	Positive attitude<br>Avg. = 2.66 (SD = 0.68)	r= –0.36***	$3.994e^{-62}$	–0.39	–0.32
	Problem solving<br>Avg. = 2.66 (SD = 0.61)	Problem solving<br>Avg. = 2.69 (SD = 0.60)	Problem solving<br>Avg. = 2.53 (SD = 0.66)	r= –0.17***	$3.603e^{-14}$	–0.21	–0.12
	Dysfunctional coping<br>Avg. = 5.50 (SD = 1.20)	Dysfunctional coping<br>Avg. = 5.46 (SD = 1.21)	Dysfunctional coping<br>Avg. = 5.70 (SD = 1.20)	r= 0.15***	$2.441e^{-12}$	0.11	0.20
	Avoidance<br>Avg. = 1.58 (SD = 0.43)	Avoidance<br>Avg. = 1.54 (SD = 0.40)	Avoidance<br>Avg. = 1.75 (SD = 0.50)	r= 0.25***	$3.710e^{-30}$	0.21	0.29
	Turning to religion<br>Avg. = 1.55 (SD = 0.80)	Turning to religion<br>Avg. = 1.58 (SD = 0.82)	Turning to religion<br>Avg. = 1.46 (SD = 0.75)	r= –0.03	0.259	–0.07	0.02
	Social support<br>Avg. = 2.38 (SD = 0.78)	Social support<br>Avg. = 2.35 (SD = 0.77)	Social support<br>Avg. = 2.49 (SD = 0.78)	r= 0.13***	$4.780e^{-9}$	0.09	0.17
		Social support, Avoidance strategies, Positive attitude, Problem solving and Turning to religion					
**BSCS**	Self-control (total score)<br>Avg. = 44.36 (SD = 8.83)	Self-control (total score)<br>Avg. = 45.55 (SD = 8.27)	Self-control (total score)<br>Avg. = 39.58 (SD = 9.37)	r= –0.32***	$1.807e^{-50}$	–0.36	–0.28
	General self-discipline (GSD)<br>Avg. = 31.32 (SD = 6.66)	General self-discipline (GSD)<br>Avg. = 32.22 (SD = 6.27)	General self-discipline (GSD)<br>Avg. = 27.68 (SD = 6.98)	r= –0.32***	$2.647e^{-50}$	–0.36	–0.28
	Impulse control (IC)<br>Avg. = 13.04 (SD = 3.07)	Impulse control (IC)<br>Avg. = 13.33 (SD = 2.94)	Impulse control (IC)<br>Avg. = 11.89 (SD = 3.30)	r= –0.23***	$1.548e^{-25}$	–0.27	–0.19
**LOC scale**	Internal LOC score<br>Avg. = 12.84 (SD = 2.17)	Internal LOC score<br>Avg. = 12.94 (SD = 2.11)	Internal LOC score<br>Avg. = 12.44 (SD = 2.39)	r= –0.16***	$7.868e^{-14}$	–0.21	–0.12

**BFI-10**	Agreeableness<br>Avg. = 6.52 (SD = 1.69)	Agreeableness<br>Avg. = 6.62 (SD = 1.63)	Agreeableness<br>Avg. = 6.07 (SD =1.85)	r= –0.18***	$1.450e^{-15}$	–0.22	–0.13
	Conscientiousness<br>Avg. = 7.25 (SD = 1.63)	Conscientiousness<br>Avg. = 7.37 (SD = 1.59)	Conscientiousness<br>Avg. = 6.75 (SD = 1.71)	r= –0.21***	$6.103e^{-21}$	–0.25	–0.16
	Emotional stability<br>Avg. = 5.76 (SD = 2.10)	Emotional stability<br>Avg. = 6.11 (SD = 2.01)	Emotional stability<br>Avg. = 4.36 (SD = 1.85)	r= –0.47***	$1.322e^{-114}$	–0.51	–0.44
	Extraversion<br>Avg. = 6.12 (SD = 1.86)	Extraversion<br>Avg. = 6.20 (SD = 1.81)	Extraversion<br>Avg. = 5.80 (SD = 2.00)	r= –0.10***	$3.113e^{-6}$	–0.15	–0.06
	Openness<br>Avg. = 7.10 (SD = 1.89)	Openness<br>Avg. = 7.05 (SD = 1.87)	Openness<br>Avg. = 7.27 (SD = 1.94)	r= 0.02	0.506	–0.03	0.06

* p<.05, ** p<.01, *** p<.001

† Participants who responded "other" to the question about the gender (n=18) were excluded from this analysis

Avg.=Average

Table S2: Item by item analysis of the PSS-10. The table reports the average score obtained in each item of the PSS-10 scale by high perceived stress participants and low perceived stress participants. The difference between the two groups has been tested through an independent sample t-test.

**Item**	**High stress**	**Low stress**					**95% CI for Cohen's d**	
	**M (SD)**	**M(SD)**	***t***	***df***	***p***	**Cohen's *d***	***Lower***	***Upper***
1. In the last month, how often have you been upset because of something that happened unexpectedly?	2.62 (0.91)	1.84 (0.91)	–27.95***	2033.00	8.802e-146	–1.57	–1.70	–1.45
2. In the last month, how often have you felt that you were unable to control the important things in your life?	3.09 (0.80)	1.60 (0.98)	–28.04***	2033.00	1.592e-146	–1.58	–1.70	–1.45
3. In the last month, how often have you felt nervous and "stressed"?	3.51 (0.60)	2.08 (0.92)	–29.48***	2033.00	2.528e-159	–1.66	–1.78	–1.53
4. In the last month, how often have you felt confident about your ability to handle your personal problems?	2.30 (0.83)	1.30 (0.78)	–22.75***	2033.00	3.224e-102	–1.28	–1.40	–1.16
5. In the last month, how often have you felt that things were going your way?	2.97 (0.85)	2.02 (0.86)	–19.82***	2033.00	4.334e-80	–1.11	–1.23	–1.00
6. In the last month, how often have you found that you could not cope with all the things that you had to do?	2.85 (1.04)	1.76 (1.08)	–18.06***	2033.00	9.960e-68	–1.01	–1.13	–0.90
7. In the last month, how often have you been able to control irritations in your life?	2.72 (0.91)	1.82 (0.85)	–18.71***	2033.00	3.646e-72	–1.05	–1.17	–0.94
8. In the last month, how often have you felt that you were on top of things?	2.93 (0.78)	1.76 (0.92)	–23.20***	2033.00	8.708e-106	–1.30	–1.42	–1.19
9. In the last month, how often have you been angered because of things that were outside of your control?	2.96 (0.84)	1.68 (0.97)	–23.958***	2033.00	6.245e-112	–1.345	–1.463	–1.228
10. In the last month, how often have you felt difficulties were piling up so high that you could not overcome them?	2.92 (0.89)	1.19 (0.91)	–33.972***	2033.00	9.779e-201	–1.908	–2.032	–1.783

* p<0.05, ** p<0.01, *** p<0.001

Table S3: List of predictors

The table reports the 18 variables that were extracted from the responses to the questionnaire and entered as perceived stress predictors in the regression models and in the ML feature selection algorithm (CFS) for classification. The last two variables are those to predict.

Being male: this is a dichotomous variable indicating the gender of the participant (1=male, 0=female, NA=neither male nor female).
Age: this is a continuous variable indicating the age of the participant.
Education: this is a continuous variable indicating years of education of the participant.
Being student: this is a dichotomous variable indicating who is a student (1=student, 0=other employment condition).
Monthly income: this is an ordinal variable indicating monthly household income. The response value ranges from 1= "less than EUR 500", 2= "from EUR 500 to 1000", 3= "from EUR 1000 to 2000", 4= "from EUR 2000 to 3000", 5= "from EUR 3000 to 4000", 6= "more than EUR 4000".
Household members: this continuous variable indicates the number of household members.
COPE avoidance: this is a continuous variable indicating the participant's mean score in the COPE-NVI-25 avoidance strategies scale.
COPE religion: this is a continuous variable indicating the participant's mean score in the COPE-NVI-25 turning to religion scale.
COPE positive: this is a continuous variable indicating the participant's mean score in the COPE-NVI-25 positive attitude scale.
COPE support: this is a continuous variable indicating the participant's mean score in the COPE-NVI-25 social support scale.
COPE problem: this is a continuous variable indicating the participant's mean score in the COPE-NVI-25 problem solving scale.
BSCS total score: this is a continuous variable indicating the participant's total score in the Brief Self-Control Scale (BSCS).
BFI-10 agreeableness: this is a continuous variable indicating the participant's score in the 10 item Big Five Inventory (BFI-10) Agreeableness scale.
BFI-10 conscientiousness: this is a continuous variable indicating the participant's score in the BFI-10 Conscientiousness scale.
BFI-10 emotional stability: this is a continuous variable indicating the participant's score in the BFI-10 Emotional stability scale.
BFI-10 extraversion: this is a continuous variable indicating the participant's score in the BFI-10 Extraversion scale.
BFI-10 openness: this is a continuous variable indicating the participant's score in the BFI-10 Openness scale.
Internal LOC: this is a continuous variable indicating the participant's score in the Internal subscale of the Short Version of the Locus of Control Scale.
PSS-10 score: this is the score obtained by the subjects in the PSS-10 scale.
Low vs. high stress: this dichotomous categorical variable indicates the low perceived stress participants and the high perceived stress participants in accordance with the cut-off of 1.5 SD above the Italian mean in the PSS-10 (low=low perceived stress, high=high perceived stress).

Table S4: Details on ML classifiers parameters

The parameters of the ML classifiers run in WEKA 3.9 software are here reported. It should be noted that these are also the default parameters automatically chosen by the software to run these algorithms.

Logistic:

- numDecimalPlaces—The number of decimal places to be used for the output of numbers in the model. = 4
- batchSize—The preferred number of instances to process if batch prediction is being performed. More or fewer instances may be provided, but this gives implementations a chance to specify a preferred batch size. = 100
- debug—Output debug information to the console. = FALSE
- ridge—Set the Ridge value in the log-likelihood. = 1.0E-8
- useConjugateGradientDescent—Use conjugate gradient descent rather than BFGS updates; faster for problems with many parameters. = FALSE
- maxIts—Maximum number of iterations to perform. = –1
- doNotCheckCapabilities—If set, classifier capabilities are not checked before classifier is built (use with caution to reduce runtime). = FALSE

SVM (SMO):

- buildCalibrationModels—Whether to fit calibration models to the SVM's outputs (for proper probability estimates). = FALSE
- numFolds—The number of folds for cross-validation used to generate training data for calibration models (–1 means use training data). = –1
- randomSeed—Random number seed for the cross-validation. = 1
- c—The complexity parameter C. = 1.0
- numDecimalPlaces—The number of decimal places to be used for the output of numbers in the model. = 2
- batchSize—The preferred number of instances to process if batch prediction is being performed. More or fewer instances may be provided, but this gives implementations a chance to specify a preferred batch size. = 100
- kernel—The kernel to use. Polykernel –C 250007 -E 1.0
- checksTurnedOff—Turns time-consuming checks off—use with caution. = FALSE
- debug—If set to true, classifier may output additional info to the console. = FALSE
- filterType—Determines how/if the data will be transformed. = Normalized training data
- toleranceParameter—The tolerance parameter (should not be changed). = 0.001
- calibrator—The calibration method to use. = Logistic
- doNotCheckCapabilities—If set, classifier capabilities are not checked before classifier is built (Use with caution to reduce runtime). = FALSE
- epsilon—The epsilon for round-off error (should not be changed). 1.0E-12

Naïve Bayes:

- useKernelEstimator—Use a kernel estimator for numeric attributes rather than a normal distribution. = FALSE

- numDecimalPlaces—The number of decimal places to be used for the output of numbers in the model. = 2
- batchSize—The preferred number of instances to process if batch prediction is being performed. More or fewer instances may be provided, but this gives implementations a chance to specify a preferred batch size. = 100
- debug—If set to true, classifier may output additional info to the console. = FALSE
- displayModelInOldFormat—Use old format for model output. The old format is better when there are many class values. The new format is better when there are fewer classes and many attributes. = FALSE
- doNotCheckCapabilities—If set, classifier capabilities are not checked before classifier is built (use with caution to reduce runtime). = FALSE
- useSupervisedDiscretization—Use supervised discretization to convert numeric attributes to nominal ones. = FALSE

Random Forest:

- seed—The random number seed to be used. = 1
- storeOutOfBagPredictions—Whether to store the out-of-bag predictions. = FALSE
- numExecutionSlots—The number of execution slots (threads) to use for constructing the ensemble. = 1
- bagSizePercent—Size of each bag, as a percentage of the training set size. = 100
- numDecimalPlaces—The number of decimal places to be used for the output of numbers in the model. = 2
- batchSize—The preferred number of instances to process if batch prediction is being performed. More or fewer instances may be provided, but this gives implementations a chance to specify a preferred batch size. = 100
- printClassifiers—Print the individual classifiers in the output. = FALSE
- numIterations—The number of iterations to be performed. = 100
- debug—If set to true, classifier may output additional info to the console. = FALSE
- outputOutOfBagComplexityStatistics—Whether to output complexity-based statistics when out-of-bag evaluation is performed. = FALSE
- breakTiesRandomly—Break ties randomly when several attributes look equally good. = FALSE
- doNotCheckCapabilities—If set, classifier capabilities are not checked before classifier is built (use with caution to reduce runtime). = FALSE
- maxDepth—The maximum depth of the tree, 0 for unlimited. = 0
- calcOutOfBag—Whether the out-of-bag error is calculated. = FALSE
- numFeatures—Sets the number of randomly chosen attributes. If 0, int(log_2(#predictors) + 1) is used. = 0
